# Supplementary material for: Compatible Consortium of Endophytic Bacillus halotolerans Strains Cal.l.30 and Cal.f.4 Promotes Plant Growth and Induces Systemic Resistance against Botrytis cinerea
Source: Biology (Basel). 2023 May 27;12(6):779. doi: 10.3390/biology12060779 (PMC10295511; doi:10.3390/biology12060779)
Supplement: Supplementary file 1 [file biology-12-00779-s001.zip › biology-2398677-supplementary.pdf]

Supplementary Material

Table S1. Primers used for RT-qPCR.

Genes	Forward Primer	Reverse Primer	Reference
Ubiquitin (ubi3)	5′-GCAGACTATAACATCCAGAAAGAG-3′	5′-AACAACAAAGCACACAGCCATC-3′	[24]
ERF1	5′- ATTGGAGTTAGAAAGAGGCCAT-3′	5′-CTCATTGATAATGCGGCTTG-3′	[26]
ACO1	5′-TTCCAGCACCAGAGTTGATTG -3′	5′-ACAGTAGTCTCCACAGCCTTC-3′	-
PR2	5′-TCCAGGTAGAGACAGTGGTAAA-3′	5′-CCTAAATATGTCGCGGTTGAGA-3′	[25]
PR3	5′-AATTGTCAGAGCCAGTGTCC-3′	5′-TCCAAAAGACCTCTGATTGC-3′	-
TomLoxA	5′-TGAACCATGGTGGGCTGAAA-3′	5′-CTGCCCGAAATTGACTGCTG-3′	[27]
